# Feasibility and Acceptability of a Smartphone App for Daily Reports of Substance Use and Antiretroviral Therapy Adherence among HIV-Infected Adults

**DOI:** 10.1155/2016/9510172

**Published:** 2016-08-16

**Authors:** Sarahmona M. Przybyla, Rebecca K. Eliseo-Arras, Gabriela Krawiec, Emily Gower, Kurt Dermen

**Affiliations:** ^1^School of Public Health and Health Professions, Department of Community Health and Health Behavior, State University of New York at Buffalo, 3435 Main Street, Buffalo, NY 14214, USA; ^2^Research Institute on Addictions, State University of New York at Buffalo, 1021 Main Street, Buffalo, NY 14203, USA

## Abstract

While substance use is one of the most consistent predictors of poor adherence to antiretroviral therapy (ART), few studies among people living with HIV (PLH) have utilized mobile phone-based assessment of these health behaviors. PLH were recruited from primary care clinics to report ART and substance use using a smartphone application (app) for 14 consecutive days. The app's feasibility as a data collection tool was evaluated quantitatively via surveys and qualitatively via in-depth interviews to assess daily report completion, compliance, and study satisfaction. Overall, 26 participants (M = 49.5 years, 76% male) completed 95.3% of time-based daily reports. Participants reported high satisfaction with the app and expressed future interest in using smartphones to report daily behaviors. High completion rates and participant acceptability suggest that smartphones are a feasible, acceptable method for collecting substance use and ART data among PLH. Potential areas of concern such as sufficient training and assistance for those with limited smartphone experience should be considered for future app-based research studies among PLH.

## 1. Introduction

Considering the standard of care for HIV treatment since 1996, combination antiretroviral therapy (ART) has resulted in widespread improvements in virologic outcomes for people living with HIV (PLH) and has yielded significant declines in HIV-related morbidity and mortality [1]. To achieve maximal suppression of viral replication and prevent drug resistance, consistently high adherence to prescribed regimens is critical. The most significant contributor to viral rebound is suboptimal ART adherence [2, 3] with extended treatment interruptions posing a higher risk of virologic rebound [4, 5]. Consequently, identifying modifiable barriers to ART adherence is a public health priority.

Substance use is particularly prevalent among HIV-positive adults. Among PLH in medical care, 66.4% report current alcohol use with more than 25% reporting at least weekly consumption [6]. Additionally, PLH have a prevalence of alcohol use disorders that is two to three times that of the general population [7]. Heavy alcohol consumption is independently linked with earlier mortality among PLH [8] and decreased overall survival of more than three years with weekly alcohol use and more than six years with daily consumption [9]. In addition, a significant proportion of PLH use marijuana; prevalence estimates range from 10 to 24% [10, 11]. The growing trend towards the legalization of medical and recreational marijuana will likely have a continued impact on the prevalence of use among PLH. Notably, substance use is one of the most reliable predictors of poor adherence to ART [12]. Research suggests that alcohol use accelerates HIV disease progression directly through interference with ART metabolism [13] and indirectly via decreased ART adherence [14]. While previous studies have generally demonstrated a positive association between alcohol consumption and ART nonadherence [15, 16], a recent systematic review has found that the relationship may be more nuanced [17]. Findings examining the relationship between marijuana use and suboptimal adherence among PLH have been mixed. Some studies show an association [18, 19] while others have found no relationship [20, 21]. While most studies have focused on unintentional nonadherence to ART in the context of alcohol or drug use, other studies have found that substance use is linked to intentional nonadherence specifically when PLH support the belief that mixing illicit drug use with HIV medications is a harmful combination [22–24]. Consequently, the effects of alcohol and marijuana use on HIV treatment outcomes have important public health implications. In particular, behavioral interventions that address substance use may improve HIV disease management and postpone disease progression.

Valid ART measures are essential to assess virologic effects of nonadherence as well as to test the efficacy of behavioral interventions to improve adherence to ART regimens [25]. Traditional tools for measuring medication adherence have inherent limitations. For example, retrospective self-reports are subject to recall errors and social desirability bias while electronic medication monitoring devices (e.g., MEMS caps) are costly and run the risk of malfunctioning [26]. Given their widespread use and convenience, the use of mobile technologies to measure both substance use and ART adherence in near real time is a promising strategy that would allow for the immediate identification of adherence challenges before the loss of viral suppression [27]. Demonstration that event-level information on the occurrence of substance use and ART adherence in one's natural environment could be collected reliably by use of a less expensive method, such as participant self-report via smartphone, would fill a known gap in the literature regarding feasibility and acceptability of such methods and may have implications for educational program development among PLH. Similar to other procedures that apply phone protocols (e.g., interactive voice response; IVR) using a smartphone-based application (app) for daily data collection minimizes recall bias, promotes ecological validity, and minimizes missing data or out-of-range responses [28–30]. Gathering data on the day-to-day experiences and behaviors of PLH has the potential to provide a unique perspective of the frequency of substance use and medication compliance in a real world setting and can reveal detailed information about social and environmental influences that may cooccur with these events. Importantly, using this participant-initiated method of data collection may be particularly appropriate for collecting data on alcohol and marijuana due to the episodic nature of substance use behaviors [31].

The emerging area of developing mobile technology for public health intervention calls for careful research among target populations to explore the acceptability of delivering such programs [32]. Questions remain about the feasibility, acceptability, and user preferences of collecting daily electronic reports of health-related behaviors among PLH. For example, stigmatization of HIV [33] may raise confidentiality concerns that may impede participation. In addition, a recent IVR study found that a significant minority of participants (20%) did not utilize the IVR system at all [34], indicating the importance of identifying preferences for engagement and utilization among potential participants. More than 90% of Americans own a cell phone with 64% owning a smartphone [35]. This increasing trend of smartphone ownership provides a potentially promising platform for delivering substance use and adherence interventions broadly and inexpensively, especially for those who may not typically access in-person interventions. While the use of mobile technologies in HIV healthcare and prevention delivery in general and ART adherence in particular is growing, previous studies using cellular phones as a technology platform most often aim to improve adherence via the use of reminders to take one's medication, most commonly in the form of text messaging [36, 37]. Few ART adherence studies assessed other behaviors that impact ART adherence, such as substance use. Therefore, the aim of the pilot study was to quantitatively and qualitatively explore the feasibility of data collection via app-based reporting of substance use and adherence to ART regimens to aid in the appropriate design and implementation of subsequent education or intervention programs tailored towards substance-using populations with ART adherence concerns.

## 2. Methods

### 2.1. Participant Recruitment and Screening Procedures

Study participants were recruited from two HIV primary care clinics that provide clinical care to the majority of PLH in the Western New York region, each being Patient Centered Medical Home (PCMH) Level 3-certified clinics. The first site was a community-based clinic and the second site was a state-certified, hospital-based Designated AIDS Center (DAC). Study eligibility criteria included being at least 18 years old, English-speaking, able to read at a Grade 7 level or better, HIV-infected, and currently on a prescribed ART regimen for at least three months. Eligibility criteria also included at least two days of alcohol use and at least one day of ART nonadherence in the past week. A two-stage screening procedure was used between June 2014 and February 2015. First, potential participants were asked to complete a brief self-administered health screening survey upon arrival at the clinic for a scheduled medical appointment. Second, research staff gave a brief study overview to eligible participants; those who agreed to participate were scheduled for a study visit.

### 2.2. Study Procedures

**Figure 1:**
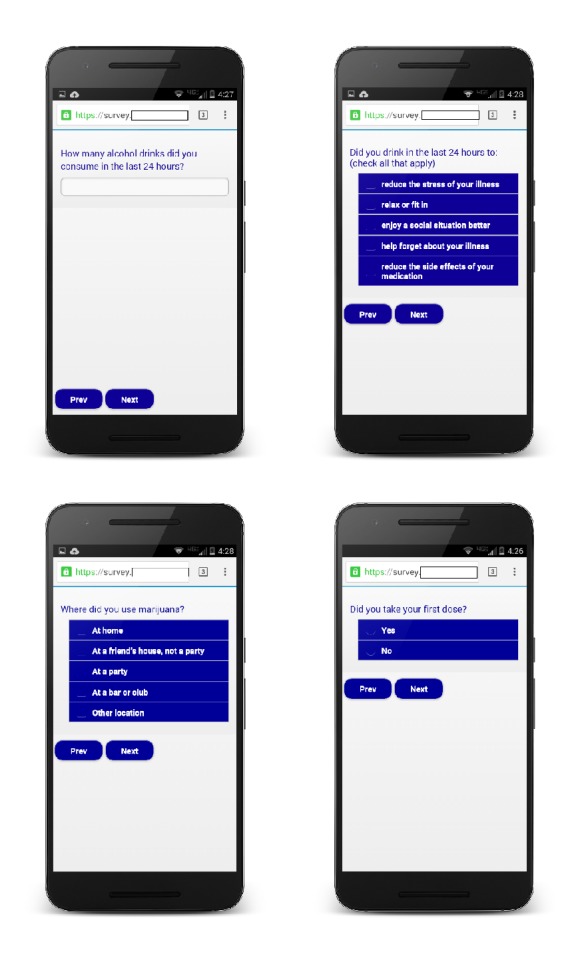
Screenshots of sample DRUM daily report questions.

Eligible participants completed informed consent and a review of study instructions at the first in-person study appointment. Participants brought their ART medication in the originally prescribed bottles or pill boxes to verify and document the prescribed regimen and completed a self-administered survey. They also received a detailed training session regarding basic smartphone operation and data entry for the completion of daily reports using the DRUM app (see Figure 1) and had the opportunity to complete practice reports in the presence of study staff members. They also received a paper-based instruction manual and were advised to contact study staff in the event of technical challenges. Participants were instructed on study policies for appropriate use of the smartphone, advised that the device was for research purposes only (e.g., to complete daily reports, contact the study staff), and informed that usage records would be monitored. For the next 14 days, participants received text message reminders at 4 pm to complete their daily reports. After the 2-week reporting period, participants completed the second in-person study appointment to return smartphones (to be reused by subsequent participants) and completed a self-administered survey. With consent to be audio-recorded, an in-depth interview was also conducted with a research staff member. Participants also received an informational pamphlet regarding substance use and ART. Compensation included $10 and $30 gift cards for the first and second study visits, respectively. Participants received $1 for each submitted daily report and a $3 bonus each week for completing seven consecutive reports (maximum compensation $20 for daily reports). Total maximum compensation for the entire study was $60. All study procedures were Institutional Review Board approved.

### 2.3. DRUM App

With the ability to operate on any smartphone platform, a mobile web application called DRUM (Daily Reports of Using Medications) was created for daily report completion. The DRUM app was developed by the principal investigator, a project coordinator, and a web-support project manager. The app was run by a browser, allowing users access as if it was a webpage. Study-issued smartphones (Motorola Droid Razr M) had the DRUM app installed on the home screen preset with a unique 5-digit passcode. While smartphone ownership was not an inclusion criterion, participants who preferred to use their own smartphone were permitted to do so and added the DRUM app on their home screen, similarly using an assigned passcode to open the app. Images of key screens and functionality are shown in Figure 1. Once text-prompted to respond, respondents were given a 2-hour window to access the DRUM app and complete the daily report (i.e., time-based reporting) to maintain fixed assessment intervals. If participants failed to complete a report by 6 pm, they had the ability to access the DRUM app and complete a make-up report the following day. The same set of closed-ended sequential questions assessed specific behaviors in the previous 24-hour period. Daily reports were designed to display one question on the screen at a time, asking participants to either check an appropriate box, fill in a number, or select responses from a drop-down menu. Navigation between questions was facilitated by the use of a “previous” and “next” button. Responses were uploaded with a time and date stamp to a secure server in real time.

### 2.4. Measures

#### 2.4.1. Visit 1 Survey

Participants completed a brief self-administered survey which assessed sociodemographic (e.g., age, marital status, and educational attainment) and clinical characteristics (e.g., date of ART initiation, viral load detectability). Other measures are described as follows.

*Alcohol Use*. Alcohol use was assessed using the AUDIT, a 10-item scale used to measure alcohol consumption and identify risks for alcohol use and dependence [38].

*Substance Use*. Use of other drugs in the last month, including illegal drugs and prescription drugs not used as prescribed, was assessed using questions adapted from a previous study of PLH [39].

*Mobile Phone Technology and Internet Utilization*. Participants completed a 20-item measure that assesses mobile phone and Internet utilization. These items were adapted from a mobile phone-based HIV prevention intervention [40] and included questions regarding utilization of phones, laptop, desktop, or tablet computers for a variety of reasons including email, text messages, and app use.

*ART Medication Regimen and Adherence Survey*. Participants completed an interviewer-administered survey that asked about one's currently prescribed ART medication regimen. Participants were queried about adherence to ART using the AIDS Clinical Trials Group (ACTG) Adherence Questionnaire, which employs a 4-day recall period [41]. Participants completed a visual analog adherence rating scale to indicate the point along a continuum showing the percentage of ART they have taken in the previous month. Standard instructions were designed to counter socially desirable response biases by acknowledging that it can be difficult to take ART [42].

#### 2.4.2. Daily Reports via DRUM App

For 14 consecutive days, participants were asked “How many alcohol drinks did you consume in the last 24 hours?” Positive responses were followed by additional questions, including the time of first and last drink, location of alcohol consumption (e.g., bar, friend's house), and reasons for drinking (e.g., enjoy a social situation better, reduce the stress of illness). Similar items assessed marijuana use. Each daily report also asked “What time did you take your first dose?” Participants who indicated that they did not take their dose received a follow-up question to indicate the main reason why a dose was missed. A similar item queried about second dose, with an option to respond with “I only take one dose per day.” (See Figure 1.)

#### 2.4.3. Visit 2 Survey

After the two-week daily report period, a 14-item quantitative survey was completed at the second in-person study visit. The survey included items about overall satisfaction with the DRUM app, concerns about privacy/confidentiality, honesty of responses in the daily reports, and likelihood of future participation in a similar smartphone-based study.

#### 2.4.4. Qualitative Interview

A brief, semistructured qualitative interview guide was used to assess participants' general reactions to daily report completion, generate feedback on their experiences with the DRUM app, and learn about previous experiences and future interests in phone-based reporting of health behaviors. Interviewers also recorded detailed observation notes immediately after the qualitative interview which were added to interview transcripts to form complete data files.

### 2.5. Data Analysis

As this was a pilot study, we were interested in assessing the feasibility of recruiting, enrolling, and retaining participants. Specifically, we examined the proportion of patients screened who were eligible, the proportion of eligible participants who enrolled, overall participant retention, the proportion of study participants who opted to use their own personal smartphone for reports, the compliance rate for daily reports, and the number of smartphones lost during the course of the study. Indicators of study satisfaction, study acceptability, previous experience with device-based behavioral reporting, and likelihood of participation in future smartphone-based studies were assessed. All quantitative analyses were conducted using Stata version 14.1.

For the analysis of qualitative interviews, two authors independently reviewed transcriptions to develop a broad understanding of reactions to study participation and identify core concepts. A detailed thematic analysis was undertaken using a deductive approach. Transcripts were repeatedly read several times and a codebook was created to delineate precise descriptions that emerged from the data. The transcripts were coded deductively, labeling sections of text based on particular domains of interest to organize the text into categories. Discussions about the coding schema were conducted and discrepancies between coders were resolved by discussion and consensus. The descriptive codes were then systematically organized into broader themes [43]. Representative quotes were retained during analyses to illustrate key findings.

## 3. Results

### 3.1. Study Enrollment

**Figure 2:**
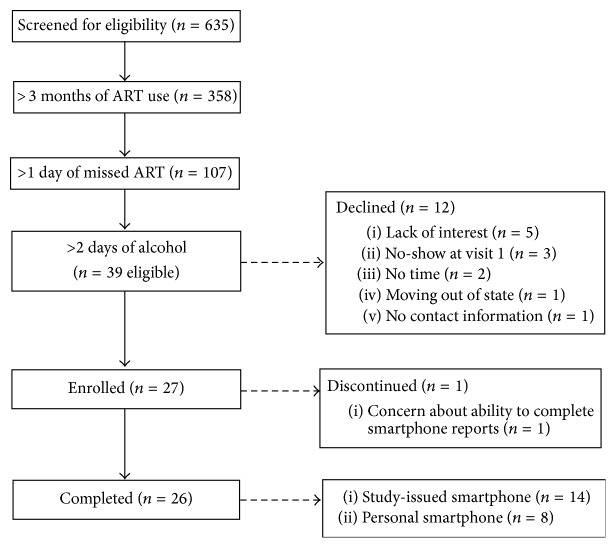
Participant recruitment and enrollment.

Across the two primary care clinic sites, 635 individuals completed the health screening survey and 39 met eligibility criteria. Twelve individuals declined to enroll (five were not interested, three initially expressed interest but failed to appear at the scheduled study visit, two did not have time to participate, one was moving to a different state, and one was missed at the clinic and provided no contact information). Of the 27 participants enrolled, one participant declined study continuation after completion of the first study visit. The current analysis focuses on the remaining 26 participants (see Figure 2). The mean number of days between eligibility screening and completion of the first study visit was 7.4 days (range = 1–28) and the mean number of days between the first and second study visits was 17.2 days (range = 15–32). The total study duration averaged 24.6 days (range = 16–47).

### 3.2. Study Participants

Sociodemographic characteristics of study participants are presented in Table 1. The majority were male (76.9%) and African American (53.8%) ranging in age from 22 to 60 years. The mean time since HIV diagnosis was nearly 17 years and most reported an undetectable viral load. Most participants (80.8%) reported past month Internet use though smartphone ownership and the use of apps on a phone was minimal. The majority demonstrated hazardous alcohol use [Mean AUDIT score = 17.08, SD = 6.56]. Additionally, nearly two-thirds reported marijuana use in the previous month.

### 3.3. Compliance with Daily Reports

There were a total of 364 possible daily reports across the 14-day period and 347 were completed (overall completion rate = 95.3%; range 21.4–100%). Forty daily reports were considered make-ups (11.4%) completed via smartphone one day after the scheduled date. Of the 347 completed reports, 92.5% were completed via smartphone. Some participants reported technical issues involving connectivity (e.g., participant was unable to access the app in a rural area) and device failure (e.g., participant temporarily misplaced the smartphone charger and was unable to complete daily reports). Upon experiencing these technical problems, participants contacted study staff resulting in 7.5% of reports being completed via telephone with a researcher. More than two-thirds of participants completed daily reports on a study-issued device. Eight participants (30.8%) opted to use personal smartphones, consistent with previous studies among PLH [44, 45]. Two devices were reported as lost and replacement phones were obtained.

During the two-week reporting period, participants reported alcohol consumption on 179 days (51.6%, range 0–14 days) with an average of 5.47 (SD = 5.68) drinks per drinking day. Marijuana use was reported on 123 days (35.4%, range 0–14 days) with an average of 3.69 (SD = 2.53) joints per marijuana use day (see Table 2). Overall ART adherence was 77.5% [(number of doses taken/number of prescribed doses) × 100]. Among the nearly three-quarters of participants who had once-daily ART regimens, the adherence rate was 87.17%. The seven participants on twice-daily regimens reported a 73.17 adherence rate (93.9% adherence rate for the first dose and 52.44% for the second dose, resp.). The most common reasons for failure to take one's first ART dose were change in daily routine (35.9%), forgetting (25.6%), and substance use (18.0%). The most common reasons for missing one's second ART dose were change in daily routine (37.5%), forgetting (17.5%), and being too busy (15.0%).

### 3.4. Quantitative Survey Findings

From a quantitative standpoint, study satisfaction was uniformly high (see Table 3). Participants had high ratings for the usefulness of the daily report training session (mean 4.64 out of 5; 92% indicating moderately/very helpful), and most (84%) indicated that the smartphone system was easy to use (mean 4.60 out of 5) despite a sizeable minority (44%) reporting limited or no previous experience with smartphones. Satisfaction with the reporting system was also high (mean 4.56 out of 5; 96% satisfied/very satisfied). Scores for accuracy regarding the level of honesty in reporting were high (mean 4.56 out of 5; 92% honest/very honest). The majority (92%) of participants indicated that they would be likely or very likely to participate in a similar daily reporting system in the future. The average time from initiation to completion of daily reports was 3.68 minutes (SD = 3.25). Most participants (80%) indicated that the survey duration was “just right,” while the remaining 20% said it was “too short” and none indicated that it was too lengthy. No participant reported prior experience with survey completion on mobile devices. Overall, participants indicated a high likelihood that they would participate in a similar daily reporting experience in the future (mean 4.48 out of 5; 92% likely/very likely).

### 3.5. Qualitative Interview Findings

Study participants reported their experiences regarding study involvement that generated four primary themes: (a) time commitment; (b) user-friendliness of the DRUM app; (c) confidentiality and privacy; and (d) key features of the DRUM app, outlined in detail below with representative quotes supporting these themes.

#### 3.5.1. Time Commitment

Participants indicated that the daily report was not a burden on their time, stating that it typically took between 2 and 10 minutes to complete reports, corroborating quantitative findings. Most indicated that the time commitment was reasonable since the survey questions were straightforward, easy to understand, and expected given the training delivered by study staff. According to one participant,

*It was not a burden on my time ‘cause I had a window period, which I took that time out to make sure I'd get my daily report in. So, I put a routine into it. I got a daily report to do. So, whatever I'm doing, I'm gonna stop. I'll be doing some drinking, we'll be smoking weed, and company at the house. I say “Hey, you know, I gotta do something. … gotta stop for a minute*.” (45-year-old male)

#### 3.5.2. User-Friendliness of the DRUM App

Many participants indicated that the study was engaging and the DRUM app was easy to use. Several also mentioned the usefulness of the training provided by study staff to adequately prepare them in their independent use of the DRUM app, which supports quantitative findings. As one participant shared,

*It's so convenient. It reminds you and then also you have in your head is what time you have to take it. So it's easier than getting a piece of paper ‘cause a piece of paper, you be like “oh, I'll do it later” And then everybody is into phones now, so it's so much easier. You have a phone with you, you can just do it. It's just simpler*. (46-year-old female)

Several similarly indicated that they “looked forward” (42-year-old male) to completing the reports as something to do during the day and that it was a “piece of cake” (52-year-old male) and “pretty self-explanatory” (48-year-old male). Other participants who reported visual issues (e.g., needing reading glasses to see smaller print) indicated that the DRUM app was “bright and clear and easy to read” (48-year-old male). As one participant describes,

*I just like it. It's more comfortable. You know, you don't have to be writing. You can just hit a screen and it goes to the answer. It's just more comfortable for me*. (48-year-old female)

For those participants who opted to use their personal smartphone, there were no accounts of difficulty in installing or using the DRUM app. Most expressed a preference for completing daily reports on their own smartphone rather than be asked to carry two devices for the duration of the study. Participants who reported no prior experience with a smartphone or those with limited technology experience stated that the DRUM app was easy to use to input their answers. As shared by one participant,

*I don't have much computer skills so it was kind of like, I feel smart. I was just scared I wouldn't know how to do it. But when you gave me the phone to take it home, I checked it, and it was more easy than I thought it was (going to be)*. (44-year-old female)

Nonetheless, some participants shared that it took some practice to become more comfortable with the number and type of questions, particularly in the first few days of the reporting period. Others mentioned that they overestimated the level of difficulty in using the DRUM app, but found it to be relatively straightforward. As described by one participant,

*To be honest, the first time it took me a bit because I was still trying to get used to it. But now I breeze right through it*. (51-year-old woman)

#### 3.5.3. Confidentiality and Privacy

Several participants described their comfort in knowing that the research study's procedures placed a strong emphasis on confidentiality. For example, the use of a unique 5-digit passcode to access the daily report system helped participants feel secure in sharing their personal health behaviors in the absence of any identifying information. They also positively endorsed the use of survey questions that did not overtly reveal their HIV serostatus. For example, one participant explained,

*Back when I was in denial (about an HIV diagnosis), I would be scared that somebody would look at my texts… Now, it didn't say “your HIV survey”. It just said, “time to do the survey”… something like that. So I felt comfortable. It didn't put me out there as an HIV-positive person but it reminded me of what I had to do*. (51-year-old woman)

Many participants indicated that they lived alone and often completed the reports in their own house or apartment with no apparent fears regarding privacy. In certain instances where reports were entered in the presence of others, participants commonly described curiosity expressed by others but did not indicate that their presence served as a barrier preventing them from accessing the DRUM app in the company of friends. As one participant described,

*Most of the time, I was at home. Only twice I wasn't. I didn't have difficulty. It was more of privacy. Like, “what are you doing?” questions from other people. It was kind of weird because I lied (about doing the report). In a sense, it was very private because the two times that it happened, the people had no understanding of what I was doing. I was just pressing numbers*. (54-year-old male)

#### 3.5.4. Key Features of the DRUM App

Two important characteristics of the daily report system were commonly discussed by study participants. First, several individuals commented on the value of the daily text message reminders. This prompt established a routine notification that cued participants to open the DRUM app. Many indicated that they had kept the phone in their pocket or in a visible location (e.g., coffee table or night-stand) making it simple to complete the reporting immediately when the text prompt was received. As one participant shared,

*I kinda enjoyed it to be honest with you, because I would be in the middle of something and my phone would buzz. I mean, I have a certain tone for texts. And I would look down after the buzz for the text and I see that it says [university's affiliation] and I say, “oh yes, I have to do this”. And I would say to my friends or whoever, “listen, just give me 2 minutes” you know. And 2 minutes is nothing*. (57-year-old male)

Second, those participants who failed to complete a report and were subsequently provided a choice to complete a make-up report the next day remarked about their satisfaction with this option. The most commonly reported reason for missing a daily report included leaving one's home and forgetting to bring the smartphone. There were also instances where changes in one's routine schedule contributed to a missed report. As one participant remarked,

*One day I did miss, but I was able to make it up the next day. And I did miss because I had just one of those days that was just… and I had the phone with me. But it was just one of those days where I was knocked out. I was exhausted and then I was still running around in between there. And I was like… by the time I realized it was 7:00. I'm like, “Oh, crap. Let me see if it's still open for me”. And no. So, it's really good (to offer a make-up report option)*. (36-year-old male)

## 4. Discussion

There is increasing interest in the use of smartphones for health behavior assessment and monitoring. Despite the growth of health-related apps available, there is limited research among PLH on user experiences and perspectives regarding the reporting of health-related behaviors using phones as the reporting platform. The results of the current study demonstrate the feasibility of reporting daily substance use and medication behaviors using mobile devices. Although no participant reported prior experience with smartphone-based health reports, after a brief training, compliance was high; more than 95% of reports were completed indicating that participants were successful at independently completing two consecutive weeks of reporting.

A primary objective of the pilot study was to demonstrate feasibility of the DRUM app. Importantly, our findings suggest that (1) simple, brief daily reports were generally found to be acceptable with high interest in future study participation; (2) offering participants the choice to complete reports on a study-issued device or a personal smartphone was well accepted; and (3) additional study features (e.g., text message reminders, make-up report options, and password-protection for app entry) were considered favorable and convenient.

Despite the general positive responses regarding study acceptability, certain challenges were identified. While technical difficulties were generally few, they were not absent. A modest number of daily reports (*n* = 26) were completed over the telephone with a research staff member rather than via the DRUM app. We listed the research study telephone number in multiple places (e.g., on the smartphone, charger, and appointment reminder card) to facilitate contact at the time of reporting in the event that a problem presented, which presumably contributed to a decreased likelihood of nonreporting as a result of technical challenges. Offering participants multiple strategies to enable communication via a variety of modalities (e.g., phone, text, or email) is strongly encouraged in subsequent studies to assist participants in adequately addressing technical difficulties when they arise.

There were two instances of study-issued devices being reported as lost and replacement phones were obtained, a finding noted in previous studies [46, 47]. The use of integrated prevention techniques such as regular, consistent monitoring of device usage is recommended for future studies. Specifically, smartphone usage (e.g., telephone calls, non-research study text messages) for each device was tracked daily by the research team. In instances that indicated an inordinate amount of nonresearch related incoming and outgoing texts and phone calls, researchers immediately deactivated the devices and contacted study participants who subsequently reported the phone was missing or stolen. In neither instance was the device recovered. For future studies, it is strongly advised to remind participants to contact research staff members immediately in the instance of a device being lost or stolen. Furthermore, participants must be reminded that engagement in unauthorized activity on a study-issued smartphone may result in device deactivation, loss of compensation for daily reports, and removal from subsequent data collection. The use of a follow-up study visit offered an opportunity to conduct additional in-person data collection but also served as a financial incentive for participants to return the devices. Additionally, future studies may consider the provision of the smartphone (excluding a data plan) after study completion as an additional incentive for study participation.

### 4.1. Limitations

Study results should be interpreted with caution given the existence of limitations. The study was conducted at two primary care clinic sites with patient screening only occurring during certain hours (i.e., 9 am–12 pm) limiting the generalizability of our findings. Our study sample was primarily middle-aged men, potentially limiting the applicability to other age groups, women, and transgendered individuals. However, the sample was representative of the HIV-positive adult population in terms of age, race, and sex for the larger geographic region from where participants were recruited. The study also had a relatively short follow-up period and daily reporting was limited to a two-hour period. Future research should weigh the potential advantages and disadvantages of the frequency and duration of reporting with careful consideration for the potential that longer reporting periods and multiple reports per day may become burdensome for participants as they experience report fatigue [47]. Additional studies should also establish the validity of reports using apps developed for research purposes.

## 5. Conclusions

This pilot study has important implications for prevention research and program development. The use of mobile devices for health assessment and intervention will likely continue to grow as smartphone ownership increases. Results demonstrated that HIV-infected adults have the capability of and interest in successfully completing health-related electronic reports, reporting positive experiences even with minimal prior exposure to smartphones. Importantly, participant satisfaction with the study procedures was uniformly positive. This data collection approach can be a valuable resource in identifying risk behaviors leading to prompt intervention in response to substance use or adherence concerns.

## Figures and Tables

**Table 1:** Demographic, clinical, substance use, and mobile technology use characteristics.

Measure	*n* (%)
*Demographic characteristics*	
Age [M (SD)]	48.4 (9.49)
Sex	
Female	4 (15.4)
Male	20 (76.9)
Transgender	2 (7.6)
Sexual identity	
Straight/heterosexual	12 (46.2)
Gay/homosexual	9 (34.6)
Bisexual	3 (11.5)
Other	2 (7.7)
Race	
African American	14 (53.8)
White	9 (34.6)
American Indian	1 (3.8)
Other	2 (7.7)
Education	
High school or less	15 (57.7)
Some college or more	11 (42.3)
Employment	
Not employed	21 (80.8)
Full or part-time	5 (19.2)
Annual income	
<$20,000	22 (84.6)
≥$20,000	4 (15.4)
*Clinical characteristics*	
Undetectable viral load	18 (69.2)
Years since diagnosis [M (SD)]	16.92 (8.65)
*Substance use characteristics*	
Cigarette smoking	
Daily	16 (61.5)
<Daily	5 (19.2)
None	5 (19.2)
AUDIT score [M (SD)]	17.08 (6.56)
Hazardous drinker (AUDIT score 8–15)	10 (38.5)
Harmful drinker (AUDIT score 16–19)	3 (11.5)
Probable alcohol dependence (AUDIT score ≥20)	4 (15.4)
Marijuana use (past month)	16 (61.5)
Crack use (past month)	7 (26.9)
*Mobile technology and Internet use characteristics*	
Mobile phone ownership	18 (69.2)
Smartphone ownership	9 (34.6)
Mean number of mobile phone numbers in past six months (SD)	1.50 (2.04)
Average number of texts sent on a daily basis^a^	
0–9	9 (50)
10–49	7 (38.9)
50+	2 (11.1)
Using apps on phone in past month^a^	11 (61.1)
Using apps on phone on a daily basis^b^	9 (81.8)
Internet use in past month	21 (80.8)
Mean number of hours per day on Internet	3.30 (2.64)

^a^ *n* = 18 participants who indicate mobile phone ownership.

^b^ *n* = 11 participants who report any app use in past month.

**Table 2:** Day-level and aggregate level substance use and ART nonadherence.

Participant	Day-level data														Aggregate level data		
	1	2	3	4	5	6	7	8	9	10	11	12	13	14	Alcohol days	Marijuana days	Nonadherence days
1	AM^N^	AM^N^	N	N	N	N	A^N^	AM^N^	AM^N^	N	N	N	N	N	5	4	14
2	AM	AM	AM	M	M	M	M	AM	M	AM	AM^N^	AM	M	AM	8	14	1
3	M^N^	M	M						M			M			0	5	1
4	A^N^		M		A		A	A	A	A	A			A	8	1	1
5			A	A		AM		A		A	A	A	A		8	1	0
6															0	0	0
7		N										A	A	A	3	0	1
8	A						A	A	A		AM				5	1	0
9	A		A		A			A	A	A					6	0	0
10	AM	AM^N^	AM	AM	AM	AM^N^	AM	AM^N^	AM	AM^N^	AM	AM	AM	AM	14	14	4
11	AM^N^	AM	AM		M	AM^N^	AM^N^			A	A		A	A	9	6	3
12	AM	M	M	AM^N^	AM	AM	M	M	AM	M	AM	AM	AM	AM	9	14	1
13	A	A	A	A	A	A	A^N^		A	A	A	A	A	A	13	0	1
14	M							M	M				A	AM	2	4	0
15		AM		AM	AM	AM		A	A	A		AM	M	AM	9	7	0
16	AM	AM^N^	AM^N^	A	AM^N^	AM^N^		AM	AM^N^	AM	AM^N^	M^N^	AM^N^	AM^N^	12	12	9
17	A		AM		AM	AM	AM	A	A	AM	AM		AM	AM	10	8	0
18	A^N^	A^N^	A^N^	A^N^	A	M	M	M	AM		M	A		A	8	5	4
19		N	N	N	N	N	N	N	N	N	N	N	N	N	0	0	13
20	AM	AM^N^	AM	AM^N^	AM	AM	AM	AM	AM^N^	M	M	AM	AM^N^	AM	12	14	4
21	A								A			A	A		4	0	0
22	AM^N^	AM^N^	M	AM	AM	M	M		AM^N^	AM^N^	AM	AM	AM^N^	AM	10	13	5
23		N		N	N	A					N	N	N	N	0	0	7
24	A^N^	A	A	A	A	A	A	A	A	A	A	A	A	A	14	0	1
25	AM^N^	A			A^N^	A	A		A	A		A^N^	A	A	10	1	3
26	M														1	0	0

A: alcohol; M: marijuana; cells with N indicate ART nonadherence.

**Table 3:** Pilot study acceptability.

Item	Rating scale (1 to 5)	M	SD
*Study team support*			
How helpful was the training you received about the smartphone system?	Not at all helpful–very helpful	4.64	0.76
My questions were answered in a timely manner by the research team.	Strongly disagree–strongly agree	4.68	0.69
*Overall reactions*			
Completing the daily reports interfered with my daily activities.	Strongly agree–strongly disagree	3.88	1.45
We'd like to know how long it took you to do the daily report. Was it	Too long–too short	2.00	0.41
How easy or difficult was it to use the smartphone system to do reports?	Very difficult–very easy	4.60	0.76
How satisfied were you with the overall reporting system?	Very dissatisfied–very satisfied	4.56	0.71
*Reporting concerns*			
How concerned were you about privacy when completing your reports?	Not at all concerned–extremely concerned	1.68	1.49
How concerned were you about confidentiality with your reports?	Not at all concerned–extremely concerned	2.00	1.58
*Willingness to participate in future studies*			
How likely would you participate in a similar daily report experience in the future?	Very unlikely–very likely	4.48	1.12
How likely would you be to recommend this study to a friend?	Very unlikely–very likely	4.32	1.11
*Reporting accuracy*			
How honest were the answers you gave on the daily reports?	Very dishonest–very honest	4.56	0.77
